# Boracic Acid Injection of Gonorrhœa

**Published:** 1880-08

**Authors:** 


Boracic Acid Injection of Gonorrhea.—Prof. J. G. Hyndman, of Cincinnati, has had remarkable success in the treatment of five cases of gonorrhœa, from injecting once or twice a day a solution of boracic acid in water, five to eighteen grains to the ounce. Two or three days was sufficient in some instances to effect a cure. He advises the five grain solution to begin with. In a female with profuse discharge, the ten grain solution cured in three days. We are rather surprised at a statement made by Dr. Hyndman that this disease is known to be more difficult to cure in persons who have had previous attacks.—*Pacific Medical Journal*

